# Information Flow between Bitcoin and Other Investment Assets

**DOI:** 10.3390/e21111116

**Published:** 2019-11-14

**Authors:** Sung Min Jang, Eojin Yi, Woo Chang Kim, Kwangwon Ahn

**Affiliations:** 1 Department of Industrial and Systems Engineering, Korea Advanced Institute of Science and Technology (KAIST), 291 Daehak-ro, Yuseong-gu, Daejeon 34141, Korea; csm0511@kaist.ac.kr (S.M.J.); wkim@kaist.ac.kr (W.C.K.); 2 Moon Soul Graduate School of Future Strategy, KAIST, 291 Daehak-ro, Yuseong-gu, Daejeon 34141, Korea; eojinkorea@kaist.ac.kr; 3 Department of Industrial Engineering, Yonsei University, 50 Yonsei-ro, Seodaemun-gu, Seoul 03722, Korea

**Keywords:** bitcoin, Granger causality, symbolic time series analysis, transfer entropy

## Abstract

This paper studies the causal relationship between Bitcoin and other investment assets. We first test Granger causality and then calculate transfer entropy as an information-theoretic approach. Unlike the Granger causality test, we discover that transfer entropy clearly identifies causal interdependency between Bitcoin and other assets, including gold, stocks, and the U.S. dollar. However, for symbolic transfer entropy, the dynamic rise–fall pattern in return series shows an asymmetric information flow from other assets to Bitcoin. Our results imply that the Bitcoin market actively interacts with major asset markets, and its long-term equilibrium, as a nascent market, gradually synchronizes with that of other investment assets.

## 1. Introduction

The Bitcoin (we use a capital B, i.e., Bitcoin, to refer to the system, and a lowercase b, i.e., bitcoin, to refer to the unit of account [1]) market, which was valued only at 0.00076 U.S. dollar (henceforth, USD) per bitcoin in its beginnings, has grown to a market capitalization of 130 billion dollars at present. At the same time, drastic market falls and rises have led to concerns and controversies. Owing to the rapid growth in market capitalization and extreme price fluctuations, legislators and economists came up with a definition of what cryptocurrency is in an economic context. Considering its potential impact on marketplaces, we investigate the characteristics of cryptocurrency as an asset, which could give us clues about cryptocurrency and its role, e.g., as a hedging instrument or its diversification benefits.

Bitcoin, which has the longest history and dominant market capitalization compared to the other cryptocurrencies, has been used in most of the literature as a representative for cryptocurrency. In particular, various studies have concurrently tried to characterize Bitcoin as a currency or commodity. Some have documented that the issuance and circulation system of Bitcoin guarantee its scarcity and lower the transaction costs in foreign exchanges [1,2]. Others have reported that Bitcoin could be used as an investment asset as a hedging or diversification tool and considerably reduce the risk of a portfolio [3,4]. However, the literature is inconclusive about its features, as Bitcoin has unique risk-return characteristics, and is volatile in a way that is unlike other assets [5,6]. For example, Yermack [7] argued that Bitcoin is neither a complementary currency nor a commodity. As such, there is a debate over the nature of Bitcoin that has yet to reach unanimous agreement.

As an extension, Erdas and Caglar [8] identified that Bitcoin only has a limited causal relation with the S&P 500 Index, and not with other assets, such as Brent oil, the USD, and the Borsa Istanbul 100 Index. Corelli [9] showed that only a few Asian currencies, such as the Thai baht and Taiwan dollar, have causal links with Bitcoin. On the contrary, a causal interconnection, namely information flow, is well identified between Bitcoin and altcoins, where the cryptocurrency market itself seems to be relatively isolated from, or independent of, other assets [10,11].

This study aims to identify the causal relationship between Bitcoin and other investment assets. In causality tests, we discover that the information-theoretic approach clearly differs from the linear autoregressive approach. Our results present strong interconnections between Bitcoin and other assets, and suggest that the Bitcoin market actively interchanges information with other markets. Conversely, a symbolized dynamic rise–fall pattern reveals an information flow with an asymmetry from other investment assets to Bitcoin. In terms of the dynamic pattern in a return series, our findings imply that the nascent Bitcoin market potentially synchronizes with other asset markets that have more trading activities and less uncertainty [12,13,14].

The remainder of this paper is organized as follows: Section 2 describes data and methodology. Section 3 reveals the results, Section 4 discusses them, and Section 5 provides the conclusion.

## 2. Materials and Methods

### 2.1. Data

Our data spanned five years, from January 2014 to December 2018, and we used daily log returns. The sample period started from the beginning of 2014 when Mt. Gox (the largest cryptocurrency exchange, handling about 70% to 80% of Bitcoin transactions by 2013) went bankrupt and the Bitcoin price collapsed [15]. The price of Bitcoin, in terms of USD per bitcoin, is provided by Quandl.com. Bitcoin exchange operates 24 h a day, and thus we chose the closing price based on that disclosed at 19:00 EST when the relevant data were updated. The S&P 500 Index is the daily closing price provided by the Center for Research in Security Prices, and gold is the international gold daily closing price (in USD) provided by Goldprice.org. USD/EUR is the exchange rate provided by the Federal Reserve Bank. Table 1 summarizes the descriptive statistics in detail.

Bitcoin has the smallest minimum and the largest maximum values among all the assets considered, thus suggesting extreme fluctuations in market returns. The kurtosis of Bitcoin is about two to three times larger than that of the S&P 500 and gold, and has the largest standard deviation in order of magnitude. These leptokurtic and fat-tailed natures further indicate that the proportion of extreme values in Bitcoin returns are quite high. In addition, negative skewness implies investors’ risk aversion attitude in the Bitcoin market as in the stock markets [16].

### 2.2. Granger Causality

Let $X_{t}$ and $Y_{t}$ be the two time series. Weiner [17] documented that $Y_{t}$ is “Causing” $X_{t}$ if we are better off in predicting $X_{t}$ using information including $Y_{t}$ than by using information about $X_{t}$ only. Especially, the Weiner–Granger causality numerically defines the concept of the causal relationship between the two variables [18]. In general, we define that $Y_{t}$ “Granger causes“ $X_{t}$ to avoid confusion by using the term “Causality” itself when there is a statistically significant regression coefficient of $Y_{t}$. The Granger causality test assumes that the following specification in vector autoregression with lag p, denoted by VAR(p), holds for two stationary time series, $X_{t}$ and $Y_{t}$:

(1) $$X_{t}=\alpha +\sum_{i=1}^{p}\beta_{i}Y_{t-i}+\sum_{j=1}^{p}\gamma_{j}X_{t-j}+\epsilon_{t},$$

where α denotes a constant term; $\beta_{i}$ presents a coefficient that quantifies the extent to which $Y_{t-i}$ explains $X_{t}$; $\gamma_{j}$ is an autoregressive coefficient that quantifies the extent to which $X_{t-j}$ explains $X_{t}$; $\epsilon_{t}$ indicates Gaussian white noise; and p represents the largest lag order obtained from the Akaike Information Criterion (AIC), Hannan Quinn (HQ), Schwarz Criterion (SC), and Final Prediction Error (FPE) [19,20,21]. The null hypothesis, that is “$Y_{t}$ does not Granger cause $X_{t}$” is defined as follows:

(2) $$H_{0}:\beta_{1}=\beta_{2}=\cdots =\beta_{p}=0.$$

### 2.3. Transfer Entropy

Transfer entropy between two variables, e.g., $X_{t}$ and $Y_{t}$ for $X_{t}^{\left(k\right)}=\left\{X_{t},X_{t-1},\;\cdots ,X_{t-k}\right\}$ and $Y_{t}^{\left(l\right)}=\left\{Y_{t},Y_{t-1},\;\cdots ,Y_{t-l}\right\}$, can be expressed as follows [22]:

(3) $$T_{Y\to X}=H\left(X_{t+1}|X_{t}^{\left(k\right)}\right)-H\left(X_{t+1}|X_{t}^{\left(k\right)},Y_{t}^{\left(l\right)}\right),$$

where $H(X_{t+1}|X_{t}^{\left(k\right)})$ denotes the degree of uncertainty for predicting $X_{t+1}$ for a given $X_{t}^{\left(k\right)}$ and $H(X_{t+1}|X_{t}^{\left(k\right)},Y_{t}^{\left(l\right)})$ stands for the degree of uncertainty for predicting $X_{t+1}$ for a given $X_{t}^{\left(k\right)}$ and $Y_{t}^{\left(l\right)}$: Both are expressed by conditional entropy. Therefore, transfer entropy $T_{Y\to X}$ can be considered to be an asymmetric measure that enables us to estimate the information flow transmitted from $Y_{t}^{\left(l\right)}$.

We calculated transfer entropy through a histogram analysis, one of the most commonly used discretization methods. Specifically, we considered a histogram defined on equally-spaced intervals (also known as bins). For a random sample, using the mean squared error, the bin width was determined by selecting an appropriate number of bins in the sample range [23,24,25]. Then, we calculated the conditional entropy of the discrete random variables [26] and finally estimated transfer entropy.

Next, we also considered symbolic time series analysis (STSA) as an alternative since it is common in various research fields, such as physics, information theory, and finance [27,28]. Based on the time-varying fluctuations in return series, STSA converts a real value into a series of symbols. First, every consecutive return was converted to binary numbers, i.e., 0s and 1s, reflecting the dynamic rise–fall pattern of the series. Subsequently, the binary numbers were transformed to a series of sequence bundles. Following Ahn et al. [28], we defined the size of a rolling window to quantify the sequence of binary numbers and then converted all of the corresponding sequence bundles into a new series of decimal numbers. Finally, transfer entropy could be obtained from the two decimal series.

## 3. Results

### 3.1. Granger Causality Test

Table 2 shows the null hypothesis and results of the Granger causality test on the basis of the bivariate VAR(p) model. Under the optimal lag order of p=1, the change of Bitcoin prices “Granger causes” the change of gold prices, but not the other way around. The change of S&P 500 and Bitcoin prices mutually “Granger caused” each other. However, regardless of lag orders, we could not find any significant causal link between Bitcoin and USD/EUR return series. We performed AIC, HQ, SC, and FPE before building up the VAR model and finally set the optimal lag order p=1. We also conducted the Granger causality test with lag order p=2 to examine the robustness of our results. We concluded that the change of lag orders does not have a significant effect on our results.

### 3.2. Normality Test

The VAR(p) model requires the residuals to be Gaussian white noise. Thus, we performed a normality test on the residuals of the bivariate VAR(p) model. Table 3 summarizes the normality test results. The test statistics of Jarque–Bera and kurtosis are all larger than the 1% critical value and that of skewness is larger than the 10% and 1% critical value for two residual series of each VAR(p) model, i.e., $M_{B,G}$ and $M_{B,S}$, respectively. As a result, we can reject the null hypothesis that the residuals of the VAR(p) model are normally distributed.

### 3.3. Transfer Entropy

**Figure 1:**
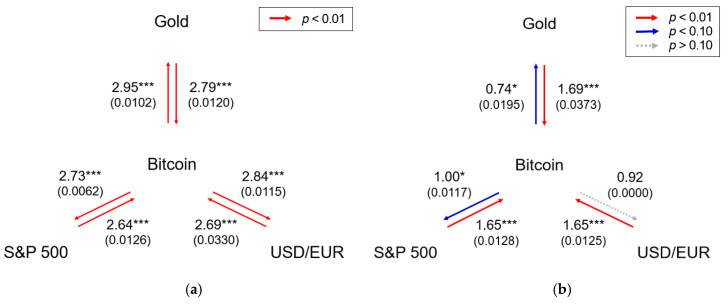
(**a**) Transfer entropy by histogram analysis; and (**b**) Transfer entropy by STSA (S=5). We display the information flow between the assets. The arrow represents the causal link, and the number indicates the estimated value of transfer entropy (the value of effective transfer entropy is also denoted in parentheses). The arrows are colored differently for each value and statistical significance. The significance level was evaluated by bootstrapping the underlying Markov process [31,34]. * and *** indicate the significance at the 10% and 1% levels, respectively.

Information flow could measure the hidden cause-effect between dynamic events [29]. We used transfer entropy to examine the causal relationship that is free from the assumption of linear autocorrelation between the two assets. Effective transfer entropy [30,31] is also considered to cope with the sample bias. Histogram-based transfer entropy presented mutual information flow, implying statistical interdependence between Bitcoin and all the other assets, as shown in Figure 1a. We estimated entropy with the bin width following Freedman–Diaconis [25] by setting k=l=1, the same condition as in the Granger causality test. Because the size of bins, related to the number of possible states of the system, could affect the entropy (amount of information [32]), we further investigated the robustness of our results with k=l=2.

We then calculated the transfer entropy by symbolizing the patterns in the return series through applying STSA, which is robust and powerful in detecting causal link between the two variables having nonlinearities [33]. The results show causal dependencies between the assets, different from the histogram-based transfer entropy, as presented in Figure 1b. The transfer entropy using STSA estimated cause-effect of the dynamic rise–fall patterns in the return series, indicating that information about the return series of other investment assets has a relatively stronger effect on that of Bitcoin than the other way around. For STSA, we set the window size S=5, trading days a week. It is also robust with the results of S=3,4,6,7. Furthermore, we set k=l=1 and k=l=2 for the same conditions as in the histogram-based transfer entropy and confirmed the robustness of our results.

## 4. Discussion

Many studies have used the Granger causality test to measure causal relationships between various time series: price fluctuations in oil and gold markets [35] and contemporaneous effects between stock returns and foreign exchange rates [36]. Earlier studies have identified a causal link of Bitcoin with gold futures [37] and with stock markets, in particular the S&P 500, in major countries [8]. Notably, there is still no empirical evidence about a causal link between Bitcoin and fiat money (e.g., the USD) in commonwealth countries except for a few Asian countries, such as the Thai baht and Taiwan dollar [8,9]. Thus, our results are generally in line with the literature; Bitcoin has a limited causal link only with some investment assets, which is asymmetric, primarily from other assets to Bitcoin.

However, as shown in Table 3, we need to pay extra attention when interpreting the results of Granger causality tests on the basis of the VAR(p) model; Jarque–Bera, skewness, and kurtosis clearly indicate that the residuals do not have a normal distribution. Although the linear autoregressive assumption allows the Granger causality test to explain intuitively the cause-effect relationship between variables; it is too naïve to explain the interactions in complex systems. It is based on correlations, which refer to a second-order statistical relationship, and so it constrains its relevance to linear systems [38]. The Bitcoin market and each investment asset market are generally considered to be complex systems, and the VAR(p) model used for the Granger causality test violates its basic assumption.

Transfer entropy, on the contrary, does not assume linear autoregression between variables, which makes it possible to test causality between non-linearly interacting variables [39]. Therefore, it has been widely used in many places to set apart driving and responding elements of the system [40]. In particular, financial markets are complex systems that express collective phenomena on the basis of the interacting individual agents [41], so transfer entropy can better detect inter-causality than the Granger causality test. For example, Marschinski and Kantz [30] measured the transfer entropy between the Dow Jones and DAX index (German stock index) and showed that there is a statistical dependency between the two. Kwon and Yang [42] analyzed stock indices between countries and documented that the United States is the dominant source of the information flow. Specifically, Pele and Pele [43] analyzed intraday log return series of Bitcoin using several econometric models and concluded that the entropy-based Value at Risk forecast provides the best results compared to the GARCH-based classical forecasts.

As shown in Figure 1a, Bitcoin mutually exchanges information with other assets and hence could serve as a hedging instrument for commodity and financial assets [3,4]. Moreover, Bitcoin even interacts with currency and is regarded as a payment platform, which supports the role of currency having many conversions and real-time exchanges with conventional currencies [1]. In particular, considering the magnitude and the statistical significance level, the dependency between Bitcoin and the others is similar so that the assets potentially interrelate with each other: Other markets are mutually coupled with Bitcoin’s. In other words, Bitcoin is more likely to be an asset that actively passes on information than is an unreciprocated information respondent.

Transfer entropy has been proposed to estimate the directionality of the coupling between dynamic systems [38], and a method such as symbolic transfer entropy is often used for reducing errors and bias correction of the numerical approximation. Figure 1b clearly exhibits that the other investment assets drive the Bitcoin market so that the symbolic pattern of the others at a given time influences that of Bitcoin’s. It further supports the hypothesis that a market with more trading activities (larger trading volume) and smaller uncertainty (less return volatility) is conducive to having a leading information discovery role than is a market with relatively smaller trading activities and more uncertainty [12,13,14]. In sum, the dynamic patterns of the return series reveal the dominance of the information discovery, and our results suggest that, in the long term, as a nascent market, the Bitcoin market potentially matures [44] and/or synchronizes with other investment assets.

## 5. Conclusions

This study analyzed the causal relationship between Bitcoin and three major investment assets. Unlike the linear autocorrelation approach, particularly the Granger causality test, histogram-based transfer entropy confirmed the existence of mutual information flow between Bitcoin and the other investment assets. STSA-based transfer entropy, however, revealed the asymmetric information flow mainly from major investment assets to Bitcoin. The results indicated that the Bitcoin market—regarded as an isolated market—indeed, actively interacts with stock, commodity, and even foreign exchange markets. Transfer entropy, according to the symbolic rise–fall pattern in the return series, further implied that the nascent and immature Bitcoin market gradually synchronizes with other markets possessing investor trading experience. Additionally, this study offered evidence that supports Bitcoin as a complementary currency and a hedging instrument beyond a speculative asset.

## Figures and Tables

**Table 1:** Descriptive statistics for daily log returns of Bitcoin, S&P 500, gold, and USD/EUR.

	Min.	Max.	Mean	Std.	Skewness	Kurtosis
**Bitcoin**	−0.27	0.25	$8.96\times 10^{-4}$	$3.85\times 10^{-2}$	−0.24	6.25
**S&P 500**	−0.04	0.05	$2.50\times 10^{-4}$	$8.35\times 10^{-3}$	−0.50	3.79
**Gold**	−0.03	0.04	$5.66\times 10^{-5}$	$8.75\times 10^{-3}$	0.15	1.83
**USD/EUR**	−0.03	0.03	$-1.35\times 10^{-4}$	$5.33\times 10^{-3}$	0.11	2.52

The number of observations is 1062 for all variables. Std., Min., and Max. are standard deviation, minimum and maximum values of each time series, respectively.

**Table 2:** Granger causality tests.

Null Hypothesis (*H*_0_)	*F*-Statistics (*p* = 1)
Gold ↛ Bitcoin Bitcoin ↛ Gold	0.04 2.83 *
S&P 500 ↛ Bitcoin Bitcoin ↛ S&P 500	2.88 * 3.24 *
USD/EUR ↛ Bitcoin Bitcoin ↛ USD/EUR	0.84 0.08

The notation “A ↛ B” denotes the null hypothesis that “A does not Granger cause B.” *F*-statistic is used and * indicates significance at the 10% level.

**Table 3:** Jarque–Bera, skewness, and kurtosis tests on the residuals of the bivariate VAR(p) model.

	$H_{0}:\;\mathrm{Residuals}\;\mathrm{Are}\;\mathrm{Normally}\;\mathrm{Distributed}$		
	Jarque–Bera	Skewness	Kurtosis
$M_{B,G}$	$2.49\times 10^{3}$ ***	7.43 *	$2.48\times 10^{3}$ ***
$M_{B,S}$	$2.81\times 10^{3}$ ***	44.19 ***	$2.77\times 10^{3}$ ***
$M_{B,U}$	$2.64\times 10^{3}$ ***	2.96	$2.64\times 10^{3}$ ***

Jarque–Bera, skewness, and kurtosis were tested using *χ^2^* statistics. * and *** indicate significance at the 10% and 1% levels, respectively. $M_{X,Y}$ is the residuals of the bivariate VAR(p) model between the two asset returns, such as X and Y, where B, G, S, and U represent Bitcoin, gold, S&P 500, and USD/EUR, respectively.
